# Stress, Dysregulation of Rhythms, and Bipolar Disorder: A Challenging Field of Research

**DOI:** 10.3390/jcm13103014

**Published:** 2024-05-20

**Authors:** Mauro Giovanni Carta, Elie Georges Karam, Giulia Cossu

**Affiliations:** 1 Department of Medical Sciences and Public Health, University of Cagliari, 09124 Cagliari, Italy; maurogcarta@gmail.com; 2 St. George Hospital University Medical Center, University of Balamand, Beirut 11002807, Lebanon; egkaram@idraac.org

Clarifying the mechanisms by which circadian rhythms regulate biology is a central issue in directing life choices in the immediate future and presents an interesting challenge for current scientific research. The importance of this research aspect is well established by the Nobel Prize in Physiology and Medicine awarded in 2017 to Hall, Rosbash, and Young, who uncovered key elements of biological rhythm systems, including those in human bodies. Specifically, their work demonstrated how the biological rhythms of the body align with the Earth’s rhythms.

The internal coordination of physiological and behavioral activities with daily environmental variations is regulated by 24-h ‘circadian’ cycles [1]. Misalignment of the circadian clock alters the sleep–wake cycle, resulting in ineffective responses to environmental cues. It has been hypothesized that the abnormal division of cancer cells is also linked to disruptions in circadian rhythms, and associations have been found between changes in circadian rhythms and some cancers [2,3], with a specific role noted for light pollution in breast cancer [4]. Dysregulation of circadian rhythms has also been identified as a risk factor for cardiovascular diseases, obesity, asthma, and diabetes [5,6]. Indeed, both internal and external signals influence the clock, but light is the most significant factor [1].

It is known that sleep rhythm is a central element in both bipolar disorders and in individuals with hyperactivity and high energy levels, even in the absence of mood disorders [7,8]. Thus, misalignment of personal and social rhythms—such as those for sleeping, eating, and social commitments and contacts, which are closely connected with circadian biorhythms—has been associated with what is called ‘the spectrum of bipolar disorders’. In a neo-Kraepelinian view, the term ‘bipolar spectrum’ refers to a pyramid of conditions. At the apex are the most severe forms of bipolar disorders, and at the base are many individuals characterized by hyperactivity, high energy, and a tendency for novelty-seeking, but without clear mood disorder connotations. In the middle, moving towards the apex, are all the vulnerability scenarios with sub-clinical symptoms that transition into mood disorders ranging from the mildest to the most severe, including depression, impulse control disorders, mood swings, anxiety disorders, personality disorders, and substance abuse disorders [9,10,11,12,13,14]. In this perspective, various temperaments could represent sub-clinical forms of bipolar disorder or simply be a risk factor for them [15,16].

There is evidence of vulnerability in the spectrum of bipolar disorders towards triggers capable of altering social rhythms and biorhythms. Light pollution has been found to be associated with a high prevalence of hypomania in megacities and may even play a role in the genesis of these disorders [17,18,19,20,21]. Socio-economic and cultural changes in current lifestyles have led to substantial modifications in light patterns, which may be one of the causes for the increase in bipolar disorders in our society, or at least for a paradoxical stability over time for such a strongly disadvantageous disease. This suggests that, at least the sub-threshold forms of the bipolar spectrum, have an adaptive connotation in modern life [20].

This perspective transcends the standard nosographic categorical approach to studying the continuum of hyperthymic traits and attitudes that can be adaptive depending on external stimuli, thus embracing a dimensional approach that can also improve the characterization of prodromal symptoms and assess symptomatology. In fact, it has been found that non-pathological personality characteristics and traits of hyper-energy/hyperactivity (without any diagnoses of mood disorders) frequently have genetic variants associated with bipolar disorder. That is, those genetic characteristics would not be typical of bipolar disorder “itself” but of hyper-energy/hyperactivity and novelty-seeking traits, including in people with the disorder as a sort of “tip of the iceberg” [22,23,24,25].

According to this approach, the onset of a case of bipolar disorder could be attributed to the convergence of genetic variants associated with hyper-energy and hyperactivity, in association with certain types of stress conditions in different life phases, particularly those of heightened vulnerability [26,27,28].

The recent pandemics and the subsequent implementation of lockdowns in several countries have inadvertently subjected people to a kind of environmental experiment, which has altered many risk factors for bipolar spectrum disorders, creating a convergence of stressors and the dysregulation of social and biological rhythms [29,30].

This framework has led to the following observations:

Older adults with dysregulation of social and personal rhythms before the pandemic and lockdown were found to be at risk for depressive episodes during the lockdown [31,32].

Stricter lockdown measures triggered more depressive episodes in people with bipolar disorders compared to less strict lockdown measures in two geographically proximate cities (i.e., Cagliari, Italy vs. Tunis, Tunisia) [33]. Healthcare workers, who were under significant pressure due to the pandemic and had more dysregulation of personal and social rhythms, were found to be at greater risk for burnout syndrome and mood disorders [34,35,36,37]. The dysregulation of social rhythms negatively impacts the course and outcomes of chronic diseases and immune responses [38,39].

The attention to the misalignment of personal rhythms related to stress has prompted reflection on the observation that individuals with a positive score on screening instruments designed for bipolar disorder, such as the Mood Disorder Questionnaire (MDQ), have been found to exhibit dysregulation of personal rhythms, particularly in relation to sleep [40,41,42,43]. In fact, the MDQ was considered useless by many researchers as a screening tool because it identified too many false positives [44,45]. Some researchers have suggested that it may be the diagnostic criteria for bipolar disorder itself that set too high a standard, and that a positive MDQ score may identify a clinically significant spectrum that is closely associated with bipolar disorder [46]. Indeed, MDQ positivity has been found to be associated with low levels of health-related quality of life, even in individuals without psychiatric diagnoses [46].

This line of research has led to the hypothesis of the existence of three different levels of hyperactivation/hyper-energy (ranging from normality to pathology) [47,48].

The first level represents an adaptive increase in energy, as observed in athletes achieving excellent results [49]. The second level is characterized by strong and prolonged stimulation of stress hormones and is identified by a positive score on the MDQ (without the individual receiving a diagnosis of bipolar disorder), as seen in healthcare workers experiencing burnout syndromes.

The third, most severe level involves hyperactivity/hyper-energy during manic episodes [47,48].

Consequently, a dysregulation of mood, energy, and social rhythms syndrome (DYMERS), corresponding to the second level of the ranking, has been proposed as a hypothesis. This perspective views DYMERS as a vulnerable condition that could potentially evolve into other disorders, with a significant risk for bipolar disorder, among others. The nature of this evolution is determined by individual-specific susceptibility based on genetic profiles and specific sensitivity to particular stresses arising from gene–environment interactions [47,48].

**Conclusions**

The scenario outlined in our discussion of current research on stress, dysregulation of rhythms, and the bipolar spectrum provides fertile ground for future investigation. This Special Issue aims to contribute to this field, offering an important step forward and serving as a stimulus for future research endeavors in the years to come. While our contribution may not provide conclusive answers at present, it undoubtedly represents a significant advancement in our understanding of these complex issues. 
